# Tubulin-Based DNA Barcode: Principle and Applications to Complex Food Matrices

**DOI:** 10.3390/genes10030229

**Published:** 2019-03-18

**Authors:** Laura Morello, Luca Braglia, Floriana Gavazzi, Silvia Gianì, Diego Breviario

**Affiliations:** Istituto Biologia e Biotecnologia Agraria, Via Adolfo Corti 12, 20131 Milano, Italy; morello@ibba.cnr.it (L.M.); braglia@ibba.cnr.it (L.B.); gavazzi@ibba.cnr.it (F.G.); giani@ibba.cnr.it (S.G.)

**Keywords:** food authentication, metabarcoding, TBP, genotyping

## Abstract

The DNA polymorphism diffusely present in the introns of the members of the Eukaryotic beta-tubulin gene families, can be conveniently used to establish a DNA barcoding method, named tubulin-based polymorphism (TBP), that can reliably assign specific genomic fingerprintings to any plant or/and animal species. Similarly, many plant varieties can also be barcoded by TBP. The method is based on a simple cell biology concept that finds a conveniently exploitable molecular basis. It does not depend on DNA sequencing as the most classically established DNA barcode strategies. Successful applications, diversified for the different target sequences or experimental purposes, have been reported in many different plant species and, of late, a new a version applicable to animal species, including fishes, has been developed. Also, the TBP method is currently used for the genetic authentication of plant material and derived food products. Due to the use of a couple of universal primer pairs, specific for plant and animal organisms, respectively, it is effective in metabarcoding a complex matrix allowing an easy and rapid recognition of the different species present in a mixture. A simple, dedicated database made up by the genomic profile of reference materials is also part of the analytical procedure. Here we will provide some example of the TBP application and will discuss its features and uses in comparison with the DNA sequencing-based methods.

## 1. Introduction

This contribution is inspired by few simple concepts that, applied to the food sector, have found ever increasing and worldwide attention since 1998 when we deposited, for patent assignment, the first version of the tubulin-based polymorphism (TBP) method, developed for plant species and varieties recognition. These concepts may be simply summarized as follows:

- Along the food chain, species identification for raw material purity assessment and food authentication is fundamental to grant products identity and quality and to protect consumers from adulterations and fraud;
- Species identification by DNA analyses are becoming widely accepted in the food sector and now many analytical tools are available;
- Among different methods, DNA barcoding has become increasingly popular and metabarcoding has attracted attention for its applications to complex food matrices.

Here, we will give an overview of DNA barcoding applications to food in comparison to fingerprinting techniques based on DNA fragment analysis.

## 2. Barcoding by Sequencing

The use of the ribosomal 16S rRNA sequence as a tool for bacterial classification and phylogenesis dates back to the late seventies, owing to the pioneering work of Carl Woese [1], when DNA sequencing was still in its infancy. Years later, the revolutionary proposal of a universal barcoding system, for the classification of all extant living species based on the nucleotide sequence of a single locus, was suggested by Hebert [2]. Since then, great effort was devoted to marker identification, primer development, testing and implementation, and to the development of integrated platforms, public databases and bioinformatics pipelines eventually coordinated by the Consortium for the International Barcode of Life (presently iBOL) [3] for data analysis. Despite some limitations, the use of barcoding markers for all eukaryotic lineages is now a generally accepted tool for investigations in a number of fields, including microbiology, ecology, taxonomy and forensics. The inexorable march of DNA barcoding keeps on producing thousands of new sequences each year, as well as hundreds of scientific papers, in addition to hundreds of new primers suitable for specific taxonomic lineages. Despite 15 years of DNA barcoding, what is clear now is that the dream of a unique barcoding region in eukaryotes will likely remain a dream since no truly universal primers will become available. In fact, target genes, one or more, and primers sets have to be carefully chosen or even designed on purpose, depending on the particular taxa, and the outcomes may vary for the different lineages. Possible uses of DNA barcoding, as well its power and limitations in the different fields of application have been widely reviewed elsewhere [4,5,6,7,8]. Therefore, we briefly summarize the essential information concerning barcoding markers that have been extensively accepted for investigations in the following different branches of living organisms:

- **Bacteria**—16S rRNA is the indisputable gold standard for microbial phylogenesis.
- **Fungi**—The Internal Transcribed Spacer (ITS) of the ribosomal DNA, including the two spacer regions ITS1 and ITS2, is the commonly accepted barcoding region for fungi [9]. The same regions are also useful for other taxonomic units, primarily for seed plants [10,11].
- **Animals**—The mitochondrial gene for the subunit 1 of cytochrome oxydase (*COI* or *Cox1*) is the generally accepted marker for almost all animal species. A huge number of primers have been designed for the amplification of COI from various animal groups (1016 COI primers in the BOLsystem database, accessed on 23 February 2019). ‘Universal’ primers amplifying the COI barcode region have also been described, but in silico analysis shows that they are poorly conserved [12]. For historical reasons, the mitochondrial gene encoding cytochrome b (*cytb*) is still used in the food sector, particularly for game meat [13].
- **Plants**—It is now accepted that in plants, universal species discrimination may never be possible with a single locus-based approach. Neither plastid data alone, nor in combination with information obtained from the nuclear genome will be able to cover all plant species. The combination of the two plastid markers, ribulose 1,5-bisphosphate carboxylase gene **(***rbcL*) and maturase K (*matK*), accepted as the core barcoding regions [14], do not grant a suitable coverage of plant species and must be often implemented with the use of other hypervariable sequences, mainly the plastid interspacer region *trn*H-*psb*A and/or the nuclear ITS [10,15]. Also, the plastid *trn*L intron have been largely applied to plant species identification [16,17]. Other plastid spacer sequences are often used for problematic taxa. Several studies have shown that about 75%–85% of plant species, up to more than 90% in some floras, can be identified at species level using a DNA barcoding approach that is based on these different combinations of markers [15].

In taxonomically problematic plant groups, where hybridization is frequent, or in lineages of relatively young age, the use of low-coverage shotgun sequencing of genomic DNA (genome skimming) could resolve genetic relationships by analyzing multiple plant barcodes at once [18,19].

## 3. General Issues about Metabarcoding: Power, Applications and Limits

The dramatic increase (400,000×) in nucleotide sequencing capacities due to the advent of next generation sequencing (NGS) technologies, and the parallel reduction in their cost, offered a significant opportunity to extend barcoding techniques to high-throughput taxon identification by metabarcoding. This is intended as a practical way to characterize whole biological communities by sequencing a common polymorphic, DNA target sequence. Less challenging than metagenomics at the data analysis level, metabarcoding of environmental samples (eDNA) has proven to be very useful to the study of extant biodiversity in microbial communities from different habitats like soil, air and water or to characterize gut microbiota [20,21].

The same strategy is increasingly applied to characterize the metazoan taxonomic composition of a wide variety of environments [20,22,23] or to characterize plant ecosystems [24] as well as to characterize animal diets from faeces, understanding food webs within ecosystems [25,26].

In principle, metabarcoding can be applied to whatever mix of biological entities, provided that a common barcode sequence is present in all the target species. Since such a universal metabarcoding marker does not exist, its choice depends on a case-by-case basis and may sometimes represent the true bottleneck. Often more than one marker must be compared in order to cover all the biodiversity present in a sample. Primer choice must also be tailored on the sample, with a preference for mixes of multiple primers to increase amplification efficiency.

Notwithstanding these limitations, several COI primer pairs have been developed specifically for metabarcoding, with a discriminatory power ranging from 73% to 100% depending on the taxon [27]. 18S rDNA or mitochondrial 12S and 16S rDNA are often the preferred target sequences [28]. Mini-barcodes have also been developed for plant species recognition but their discriminatory power is very low, so that full-length DNA barcode regions could outperform shorter markers for surveys on plant diversity in soil samples [24]. The p6 loop of the *trn*L intron has also been used in animal diet metabarcoding [29,30] and can effectively be used to identify commonly edible plant species in food [17]. ITS2 has also been used for plant metabarcoding in food [31].

The greatest advantage of DNA metabarcoding is its ability to simultaneously identify multiple taxa within complex multi-ingredient and biodegraded samples, where the application of DNA barcoding and conventional analytical methods may find considerable limitations.

On the other hand, the main limitations of DNA metabarcoding relate to restrictions in marker choice that are even higher than those for barcoding. Primers must be accurately chosen to be effective across different taxonomic units, while the amplified fragment must be shorter to cope with the DNA degradation of complex matrices. At the same time, metabarcoding primers should encompass sufficient variability to allow discrimination between MOTU (molecular operative taxonomic units). The availability of comprehensive reference databases for any target locus is also necessary.

Transforming raw read data from NGS platforms into a list of taxa is not at all a trivial task and requires complex bioinformatic skills to grant accurate quality assurance, throughout all sequence processing steps and in final data interpretation. Several variables related to the methodological framework, such as sequencing platform, filtering options, quality thresholds, chimera removal and clustering thresholds can deeply influence the outcome [32]. While there are many bioinformatic methods available for the analysis of metabarcoding data, the discriminating power of these methods is directly related to prior choices on the barcode marker and reference database composition [8]. Literature demonstrates that inappropriate marker choice and inaccurate data analysis may lead to erroneous conclusions [5].

In conclusion, DNA metabarcoding sacrifices a higher power in species discrimination, due to its tremendous capacity of analyzing a huge amount of sequence data, thus giving a chance to attain information from very complex samples often characterized by the presence of highly degraded DNA. In many ecological studies, it is not so important to define communities at the species level, but resolution at the family or genus level is sufficient to retrieve important ecological information.

### Food Metabarcoding

Food authentication is fundamental to protect consumers from common frauds such as adulteration and/or mislabeling and to comply with religious or ethical demands from the consumer. While barcoding is becoming widely applied to the authentication of single edible species, from medicinal plants to fishes [33,34,35,36], the application of metabarcoding to complex food analysis is still limited [8].

Compared to environmental samples, the food field of application would seem easier to face since the number of species is limited to hundreds, at the most. Conversely, the need to precisely assign barcodes to species level is more stringent. In accordance with this, barcoding markers and primers that need to be carefully selected are often dependent on the product type. Different metabarcoding strategies have been applied to specific sectors of food analysis. One is that of dietary supplements, like herbal products and traditional medicine. Adulterations of these products are very frequent and often occurs in the first stages of their value chains. 16S rRNA, tRNL p6-loop and nrITS2 have been used as targets for plant metabarcoding in the analysis of Traditional Chinese Medicine (TCM) products revealing a high level of species substitutions that opens the way to legal issues and health safety concerns [31,37]. More recently, herbal supplements reported to contain *Hypericum perforatum* and *Veronica officinalis* were investigated by Polymerase Chain Reaction (PCR) amplification using nrITS primers. Out of 78 *H. perforatum* herbal products only 68% contained the declared target species [38] (Raclariu et al., 2017). Only 15% of investigated *Veronica* herbal products really contained *Veronica officinalis*, whereas the main known adulterant, *Veronica chamaedrys L*., was detected in 62% of the analyzed samples [39].

Of late, different strategies for seafood metabarcoding have been developed and tested on different NGS platforms. Seafood is a sector where a large amount of substitutions and fraud has been widely reported. Some investigations were based on the use of the canonical animal *COI1* and *cytb* barcodes, or derived mini-barcodes, and were applied to species identification in specific market products. A survey on processed cod products purchased from supermarket and fast food outlets in Brazil revealed a mislabeling rate of 41% [40]. Kappel et al. [41] set up a protocol capable of identifying different tuna species in mixed samples, with a limit of detection as low as 1% (*w*/*w*). The presence of multiple undeclared species in canned tuna fish sold in the European market, not allowed by European Union (EU) laws, was occasionally found. The16S rRNA fragment was instead chosen as the DNA target for the identification of fish and cephalopod components of Surimi samples sold in the EU market [42].

Bertolini et al. [43] used the next generation semiconductor-based sequencing technology (Ion Torrent) for the identification of DNA from meat and poultry. Three different primer pairs, based on both mitochondrial rRNA sequences, 12S and 16S, were tested on DNA pools made from meat of 11 mammals and birdspecies as well as from humans and rats as possible contaminants. The strategy gave encouraging results, although amplification biases could limit detection of avian species that were less efficiently amplified than mammalian species and, therefore, underrepresented in the final data analysis. The two minor DNA species in a DNA mix of seven, present at 10% and 2%, respectively, could be efficiently determined with one out of three primer pairs, while the other two hardly detected horse DNA even at 10% level. The authors conclude that the sequencing of the products obtained from different universal PCR primers could be a useful strategy to overcome these problems of amplification.

Very recently, Dobrovolny [44] presented a DNA metabarcoding method that allows the identification and effective discrimination of fifteen mammalian and six poultry species in foodstuffs. The method, developed on the Illumina MiSeq^®^ platform, targets the same mitochondrial 16S rDNA region reported by Bertolini et al. [43], but uses, in a duplex assay, a newly designed primer pair for poultry in combination with the primer pair specific for mammalian species. It was successfully tested on 20 ternary DNA extract mixtures and four model sausages with mixtures of up to four meat types with a minimum content of 5% *w*/*w*.

A metagenomics approach, named Allfood seq, based on untargeted deep sequencing of foodstuff total genomic DNA performed with random primers, has been also proposed [45]. Tested on reference sausages, made up by different combinations of mammalian and avian meat, was able to accurately identify species at the 1% discrimination level via a read counting approach and to detect possible contaminants from all kingdoms of life. However, unexpected results, such as the putative presence of whale or monkey DNA in the tested samples, revealed the extreme importance of a correct database of reference and the need for experts in BLAST (Basic Local Alignment Search Tool) data interpretation. This holds true also for all metabarcoding data.

Quantitative assessment of relative species abundance based on sequence read numbers, which would have great impact in the food sector, is not reliable. Despite some attempts, too many variables considerably impact on the number of obtained sequence reads [8]. Therefore, DNA metabarcoding data remains, at present, solely reliable if used for qualitative evaluation.

Considering that food for human consumption includes a rather limited amount of species, over the 10–15 million estimated to populate the planet, simpler alternatives to sequence-based metabarcoding systems, depending on fragment analysis rather than NGS sequencing, should be regarded as an amenable and valid alternative (see Section 4).

## 4. Barcoding by Fragment Analysis: When Less Is More

DNA fingerprinting based on fragment analysis is the most ancient of DNA-based analytical approaches, dating back to pre-PCR times, with restriction fragment analysis (RFLP) in plants, later followed by PCR-based molecular markers (RAPD, AFLP and SSR) [46]. These markers are based on the generation of random fragments and, widely applied for genotyping at subspecies level and QTL (quantitative traits loci) mapping, they were not developed for discrimination at species-level. Nevertheless, some of these techniques have also been applied to food analysis, for species and variety identification [47]. In addition, several molecular markers targeting intron length polymorphism have been subsequently developed for genotyping or for gene mapping and marker-assisted selection especially in plants [48,49].

However, methods such as quantitative PCR (qPCR) or high-resolution melting PCR, based on highly sensitive species-specific tools, have progressively become the most preferred approaches for food authenticity [50,51]. These approaches allow the detection of the targeted species in admixtures with a low multiplexing ability that is limited by the number of fluorescence channels available.

Intron length polymorphism is exploited by two alternative barcoding methods that, based on fragment analysis, yield species distinctive fingerprinting in food: TBP [52] and Species Identification by Insertions/Deletions (SpinDel) [53]. Both methods rely on the detection of short insertion/deletion mutations in non-coding regions rather than on nucleotide substitutions. The generation of numeric profiles from fragment length information supports a high level of discrimination because a huge number of combinations is obtainable with just a limited number of loci and related alleles.

### 4.1. Species Identification by Insertions/Deletions (SpinDel) Profiling

The SpinDel protocol, developed by Pereira [53] uses a conventional genotyping methodology similar to that employed with short tandem repeats (STRs), involving multiplex PCR followed by fragment size determination done by capillary electrophoresis (CE). Thus, each species can be defined by a numeric barcode generated as a unique numeric profile of amplicons resulting from the combination of the length of indel-rich regions. The SPInDel targets the mitochondrial rDNA, amplifying short hypervariable DNA fragments interspersed within highly conserved domains. Number and position of the hypervariable regions, and suitable primers, must be chosen on purpose, depending on the taxonomic group. A computational platform for data analysis, accompanied by a collaborative online workspace, including a dedicated workbench to assist alignment of target sequences, selection of informative hypervariable regions, design of PCR primers and statistical validation of the species identification process, was made available [54,55]. Mainly developed for forensic purposes, it is appropriate for problematic samples that also contain highly degraded DNA. Degenerated primers, targeting six hypervariable regions, were developed for the simultaneous identification of humans and nine most common domestic mammalian species [53]. A validation study was performed on various type of biological material (blood, buccal swabs) and on highly processed food products, including the identification of species from mixtures. A similar high level of species discrimination is attainable in all taxa of life by designing suitable primers.

Of recent, the utility of the SPInDel concept was also extended to the identification of plants, targeting suitable combinations of variable-length sequences of the chloroplast DNA [56].

### 4.2. Tubulin-Based Polymorphism (TBP) Barcoding: The Principle

**Figure 1:**
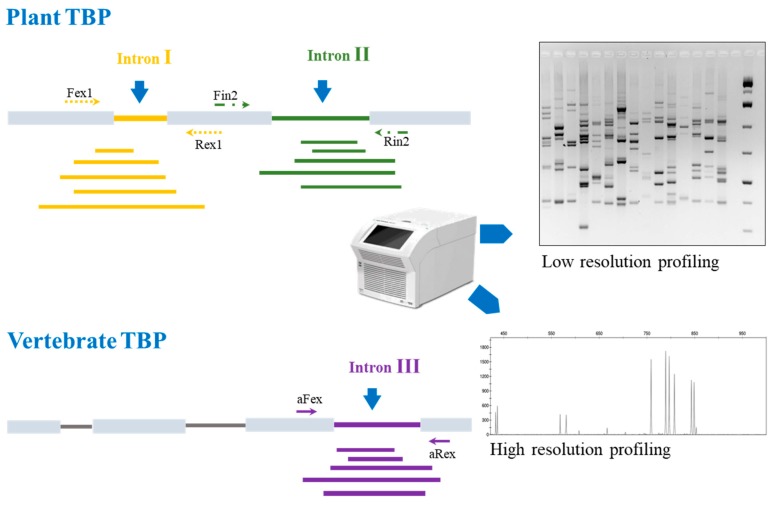
Graph reporting the principle and the output of the tubulin-based polymorphism (TBP) method. The two versions, applicable to plant and vertebrate organisms are shown with their reciprocal sources of intron length polymorphism. Amplified fragments obtained by polymerase chain reaction (PCR) can be resolved either by gel electrophoresis (bands) or by capillary electrophoresis (peaks).

Tubulin-based polymorphism relies on the unique feature of beta-tubulin genes to maintain a rigorously conserved exon-intron structure across high range taxa: this holds true for the *Embryophyta* subkingdom, on the plant side, including all land plants starting from *Bryophyta*, and for the whole Vertebrates subphylum on the animal side. Instead, most unicellular eukaryotes, like microalgae, fungi and the many different lineages of protists, bearing just one or two beta-tubulin loci, show variable and divergent exon-intron organization [57]. Conserved intron position combined with the presence of similarly conserved nucleotides in the flanking exons, coding for fundamental protein domains, allowed the design and implementation of slightly degenerated, universal primer pairs for both Embryophytes and Vertebrates. In particular, two primer pairs tag the first and second intron of plant beta-tubulin genes, while the third intron is found to be suitable for vertebrates (Figure 1).

The second, fundamental feature of a beta-tubulin based marker is that of being multilocus: in fact, the beta-tubulin family of each multicellular eukaryotic species is made up by a variable number of paralogs, all tagged by the universal primers to which some pseudogene may also add. In fact, when primer target sequences are retained, pseudogenes equally contribute to tubulin-based polymorphism, as reported for wheat [58]. The source of polymorphism is therefore dependent on both the loci number and the variability of intron length. Finally, the marker is codominant, meaning that alternative allelic variants can be detected at once. The meaning of the word allele in this case is limited to the presence of indels, since nucleotide sequence polymorphism cannot be detected. Tagging both plants’ introns increases the number of available markers, allowing higher resolution.

One of the pre-requisite of a reliable barcoding marker is the existence of a gap between the intraspecific and the inter-specific variability. This holds true also for TBP barcoding since, in our longstanding experience, intraspecific variability, when present, is generally limited to few, moderately polymorphic loci. However, as found for classical barcoding, the TBP barcoding gap is likely to be variable across taxa, being related to their specific evolutionary history [59]. While not impairing species identification, low-level intraspecific polymorphism, allowed in some case discrimination among crop cultivars.

Remarkably, grape and olive, major crops since long cultivated by agamic propagation to preserve their important agronomic traits, represent two of such cases, where intraspecific variability was high and easily documentable. In both cases, TBP was a very useful tool to discriminate at the cultivar level. Unweighted pair group method with arithmetic mean (UPGMA) dendrograms based on Nei and Li’ genetic distance calculated on the TBP data of 37 accessions of the genus *Vitis*, including different species, rootstocks, subspecies and cultivated clones, correctly grouped them in different clades and branches as similarly found with the use of internationally validated SSR markers [60]. Likewise, a consistent if not better classification, than that obtained with SSR markers, was also obtained by plant TBP genotyping of 15 different olive cultivars was obtained [61].

In plants, the combination of the two target introns (cTBP) [62] provides a number of markers sufficient for taxa discrimination at the species level. Based on the relevant number of plant species so far investigated, more then 200, where 100% success was scored for both primer annealing and species discrimination, TBP mediated plant genotyping should deserve a better consideration as a solid and reliable genotyping approach.

Discrimination at species-level within the same genus was also successfully applied to problematic genera, with loose species boundaries. Within the genus *Triticum*, characterized by high interspecific hybridization rates combined with a pronounced polyploidization, TBP was able to fingerprint three closely related *Triticum* species: *T aestivum, T. monococcum, T. spelta* and two subspecies, *T. durum* ssp. *dicoccum* and *T. durum* ssp *durum* [58].

Similarly, when applied to the genus *Citrus,* which is characterized by conflicting classifications [63] and difficult resolution at the species level by classical barcoding markers [64], the TBP analysis generated species-specific amplification patterns for all the twelve analysed *Citrus* species [65].

A newly developed version of TBP applicable to animal species has been recently developed, with a focus on edible vertebrate species. It has been successfully tested on a more restricted number of species than plants, limited to farm mammals, poultry and about 30 fish species [66].

The number of beta-tubulins in vertebrates is basically variable between six and eight, but more loci are present in some lineage due to tandem gene duplication and possible pseudogene conversions [57]. In accordance, a smaller number of amplicons was scored in meat species compared to plants. Conversely, the number of target beta-tubulin loci was found much higher and variable in fishes, compared to the other vertebrates, because of the frequent occurrence of polyploidization events [67]. Here we present a demonstrative survey of experimentally tested applications of TBP barcoding (TBB), focused on the concomitant identification of multiple species in products of the agro-food sector.

### 4.3. Reference Database and Bionformatic Needs

Given that the availability of a large set of reference sequences is needed for the successful application of any DNA barcoding classification, TBP profiles could in principle be predicted from those species for which genome sequencing data are available in order to produce a cross-referenced database. However, the often incomplete nuclear genome, even for those genomes that were first sequenced, makes any in silico prediction not fully reliable. In example, the number or beta-tubulin loci in humans has been recently revised due to the discovery of a new locus in a telomeric region [57]. Moreover, the application of TBP to the genotyping of grape cultivars revealed the presence of an additional allele in the *Vitis vinifera* cv. Pinot Noir [60], the same cultivar sequenced by the International Grape Genome Program, not duly reported in the corresponding database [68]. In fact, a minority of the genome sequencing projects can adequately cover the presence of alleles. Without the need for sequencing, a working TBP database for both meat and plant food authentication can be built easily where needed by the parallel amplification of suitable reference DNA obtained from commercially available species. Nevertheless, a large public dataset of reference profiles, also accounting for intraspecific allelic variants, could be helpful for the identification of unknown components and highly desirable for the fish sector.

## 5. TBP Application’s Survey

### 5.1. Plant Species Identification and Raw Material Purity

In simpler cases, often corresponding to the analysis of wild herbaceous accessions from pastureland, or trees from public garden areas, TBP-based PCR amplification followed by conventional agarose gel or polyacrylamide gel electrophoresis (PAGE) can be sufficient to assign specific genomic fingerprinting [69]. This could be also applied to the authentication of ornamentals plants such as cultivars and hybrids [70], or to misclassification of seed bank collection [71]. This was the case for one seed bank accession of *Camelina sativa*, whose divergent TBP profile in comparison to many others, has encouraged further karyological investigations that have recently led to the definition of a new species [72]. Such analysis can be performed in as little as one day, from DNA extraction to gel analysis, in a minimally equipped laboratory of molecular biology.

If closely related species have to be analysed, capillary electrophoresis (CE) separation should be applied to bring resolution down to the 1–2 nucleotide level. For example, TBP profiling assisted by CE was able to give unique profiles for each of the most common cereal species (barley, oat, rye, maize, etc.) and even to distinguish species within the same *Triticum* genus. The assay was applied in the food sector, to cereal flour authentication and purity assessment, and was also effectively tested on dry pasta samples [58,73], where cases of mislabelling and replacement were revealed. Suitable primers, targeting a subpopulation of cereal tubulin loci with short introns, successfully performed even in baked products, containing highly degraded DNA [58].

### 5.2. Feed Composition by TBP Metabarcoding

Feed composition is relevant for a correct balance of nutrients supplied with the diet but fraudulent admixtures or substitutions in order to reduce cost and increase profit can occur. Moreover, the presence of toxic plants must be avoided. In some case, the absence of specific plant in the diet is important for the undesirable flavor they may contribute to milk (i.e., rapeseed). Within the framework of the European Commission (EC)-funded Feed-code Project, Grant Agreement No. 315464, the TBP method based on CE resolution was implemented on an integrated analytical platform and applied to compound feed authentication [74].

TBP is per se a metabarcoding technique, since multiple loci are amplified at once (TBB) [75]. The possibility of using it in complex mixtures is therefore feasible, but limited by the complexity of the pattern that may present peak overlaps, when a very high number of species is present. When applied to the determination of the plant composition of feed raw materials and commercial cattle feed samples, CE-TBP generated mixed profiles from which the identity of each of up to 11 plant components in one feed sample could be determined [74].

**Figure 2:**
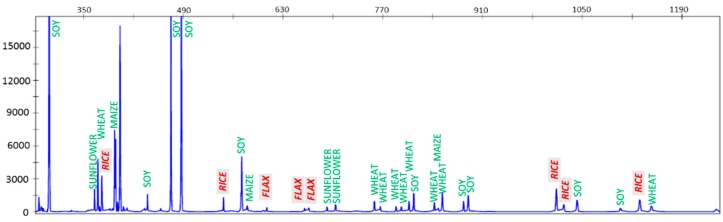
Feed sample analysis. The capillary electrophoresis (CE)-TBP profile gained from one cattle feed sample. The peak size (base pairs) is reported on the x axis and the fluorescence intensity (RFU, relative fluorescence unit) on the y axis. Green labels indicate specific peaks generated by DNA amplification of declared ingredients (sunflower, wheat, maize and soy), red labels indicate amplicons originated from detected undeclared species (flax and rice in gray boxes).

As shown in Figure 2, recognition of the different components is based on the detection, in the CE electropherograms, of diagnostic species-specific peaks, corresponding to amplified fragments that show unique sizes within the investigated dataset. Even though feed pellets, the most common type of diet supplement, are prepared under high temperature and pressure treatments, the TBP-based platform has always been able to correctly amplify fragments in the range of the analytical method (up to 1200 bp), with the only exception materials such as insilates and beetroot pulps, subjected to extensive fermentation processes. The limit of detection, on a *w*/*w* base, although variable in relation to the number of plant components and possible primer biases, was in most cases around 1% *w*/*w* in reference mixes. In a survey conducted on more than 100 feed samples tested, collected from different EU countries, 60% resulted in not conforming to the label.

Since the newly developed vertebrate primers give no amplification products in any plant assay, their use to exclude the presence of fish flour in animal feed is possible, although not tested, yet.

### 5.3. Meat and Fish Food Authentication

TBP-assisted genomic profiling of the most common meat species used in food manufacturing and a couple of possible contaminants (mouse and human) revealed that identification at the species level can be straightforward with the use of a primer pair capable of amplifying the third beta-tubulin intron commonly shared among vertebrates. By contrast with what often observed in plants, intraspecific TBP-dependent polymorphism is almost absent in mammals (different bovine and goat breeds tested), while a few allelic variants were detected in chicken, with any obvious consequence on the robustness and the integrity of the analysis. Animal TBP has been successfully tested on several DNA and meat mixtures, made up by four different species and containing down to 1% of a single species in the admixture [66]. This diagnostic ability was also confirmed in cooked or frozen/defrosted admixtures with little loss of resolution in the former, limited to the disappearance of fragments longer than 900 nucleotides.

**Figure 3:**
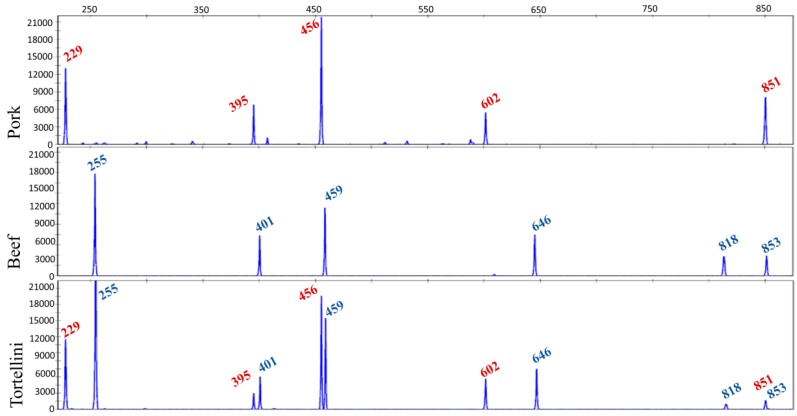
Meat-filling authentication in a sample of tortellini bought in the market. The TBP profile found in tortellini is compared with the pork and beef reference profiles. Numbers in the electropherograms refer to the length of the TBP-amplified fragment (peak size).

Finally, the TBP assay has been applied to a wide variety of processed industrial food products, available in the market. Figure 3 shows one of these applications done on a sample of tortellini where the meat filling was made with pork and beef.

This analysis on food products available in the market turned out to be very useful for recognizing their actual composition and estimate the level of reliability of what declared in the label.

The same primer pair used for meat authentication was also successfully assayed in fishes. The seafood sector is particularly challenging since the number of edible fish species worldwide is much higher than that of terrestrial animals, with the presence of many closely related species. Fish is also frequently sold as fillets, slices or hamburgers, hindering the application of classical morphological identification approaches. Therefore, the sector is particularly prone to fraud and mislabeling, as reported many times in many countries [76,77,78] and confirmed by the surveillance actions promoted by important institutions such as United Nations Food and Agriculture Organization [79] and the European Commission [79,80].

**Figure 4:**
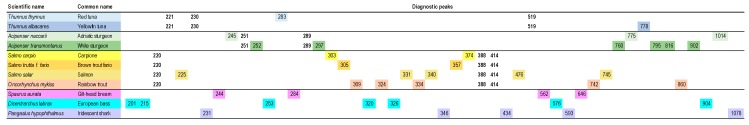
Numerical TBP profiles of representative fish species. The graph reports with colored background those TBP amplifiable fragments that specifically associate to the fish species enlisted on the left. Amplicons, retrieved from the complete TBP-based genomic profile, are denoted by their sizes, expressed in base pairs. Numbers with no background are shared within a member of a higher taxonomic ranks.

As mentioned above (Section 4.2) and with reference to terrestrial animals, the level of species discrimination of TBP in fishes is potentially higher, due to the presence of a higher number of target loci. Commercially important, closely related species such as yellow finned and red tuna, or different sturgeons or trout species can be easily recognized. As for any other TBP application, the basis of such recognition is dependent on the presence of unique diagnostic peaks corresponding to fragments only amplifiable from a single species (Figure 4). TBP primers targeting crustacean or mollusk tubulin introns could also be developed in the future.

## 6. Authenticity Testing along the Food Chain: Advantages and Limits of Sequence-Based and Fragment-Based DNA Metabarcoding

The main problem encountered by any DNA analysis when facing complex samples such as food or environmental specimens, is DNA degradation. This inconvenience can be solved by metabarcoding, choosing short target regions (100 bp) that, on the other hand, can be insufficiently informative at the species level. TBP targets cover a wide range of amplification products length, from 200–300 to 1200 nucleotides, scorable by CE with 1–2 nucleotide resolution. However, due to its multi-target features, the chance of generating enough short amplicons capable of discrimination even in presence of partially degraded DNA recovered from food remains high. The simultaneous amplification of multiple loci considerably increases the discriminatory efficiency of the procedure by avoiding null responses in cases of non-amplifiable loci, a severe limitation of methods relying on singleplex PCR. The use of a second TBP primer pair in plants enhances the sensitivity of the assay because it further increases the number of target loci. The main advantage of the TBP barcoding is full applicability to very large taxa, without need for prior knowledge of genome sequence information or target or primer set choice. In the food field of application, the TBP method presently allows, with the use of just three primer pairs, the identification of all cultivated and edible plants and most of the edible animal species, including fishes. Another advantage is the easy mode of application that relates to both the wet-lab phase, just one PCR reaction followed by capillary electrophoresis, and the data analysis. A possible technical drawback is represented by primer competition that can produce amplification biases favoring the prevalence of some amplicons over others. Although this limit could have some relevance when analyzing complex mixtures, it is generally counterbalanced by the multiplicity of the target loci. Possible PCR artifacts, such as heteroduplex formation and stutter peaks generated by polymerase slippage in homopolymeric regions, were rarely encountered and profiles obtained from species whose genome sequences were available in public databases almost perfectly matched the number and length of the amplified fragments [58,81]. On the other hand, the wet-lab phase for metabarcoding sample preparation needs several manual steps, such as library preparation, is not suitable to automation and requires accurate control of DNA quality. Sequencing data are also prone to errors that may be introduced either by PCR amplification or by sequencing, only partially solved in the analysis step. Severe primer biases may also strongly impair the detection of all the taxa present in a sample, when using a single barcoding target.

With respect to data analysis, TBP fingerprinting applications, not based on sequencing, do not require any bioinformatic skills for data analysis. The evaluation of the data quality and the recovery of signal intensity (allele size assignment) from the electropherograms is entrusted to the GeneMapper software, included with the genotyping instrument (Genetic Analyzer, Thermo Fisher Scientific, Waltham, Massachusetts, U.S.), long adopted in different fragment analysis applications [82,83,84]. The numerical data output provided by the software can be conveniently converted in an Excel spreadsheet for downstream analysis, that can be simply performed by numerical profile comparison. Even more complex profiles, such as those obtained from mixed samples or fishes, can be resolved by eye although a dedicated analytical software is under implementation to speed up the analysis. This will be of help for data normalization, background subtraction and profile comparison with a reference database of choice by similarity matching. This is particularly important for the analysis of large datasets and mixed profiles or to infer the identity of unknown species from their TBP fingerprint. Evidence for the reliability and repeatability of the fluorescent capillary electrophoresis technique, in the fast and accurate screening of indels from multiple amplification products has been provided by different authors [85,86]. Even if the TBP sensitivity level is not particularly high, it is perfectly adequate for the current detection limits requested for food admixtures and likewise comparable to that reported for food metabarcoding [41,43].

**Figure 5:**
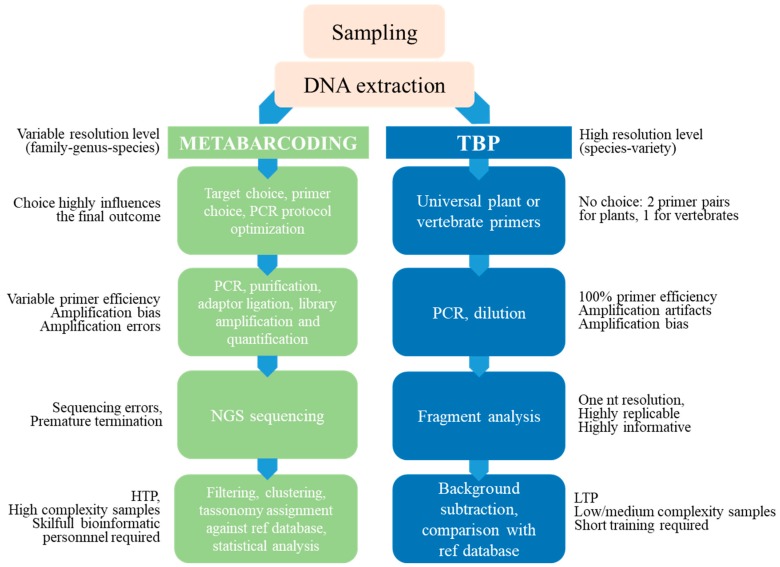
Comparison of barcoding and TBP workflows. Summary of the strength and the weakness of the classical metabarcoding method compared with TBP fingerprinting.

In conclusion, while extremely powerful for basic research purposes, metabarcoding still appears too highly demanding for routine analysis due to the difficulties in protocol standardization, the need for a challenging bioinformatic analysis, performed by highly trained people, and the availability of powerful computing facilities. Although costs per analysis on NGS platforms are constantly decreasing, thanks to fast technical advances in DNA sequencing and the availability of bioinformatics tools, they still seem too high for analytical purposes in the food sector and do not take into account the time required for data processing. This said, sequence metabarcoding could surely be recommended as the strategy of choice when dealing with particularly problematic samples, like baked or fermented products or plant extracts, characterized by a relevant level of DNA degradation but barcoding by fragment analysis offers a valid alternative in most cases. Figure 5 summarizes the strength and the weakness of the classical metabarcoding method compared with TBP fingerprinting. The comparison does not look unsuitable since TBP emerges as a competitive technique for several not irrelevant operative aspects.

## 7. Patents

Reported TBP experiments were based on the application of two of two different European patents: No. 1,144,691 (expired in 2017), for plant species genotyping and N.3011049 applicable to animals.
